# Study on Impact Acoustic—Visual Sensor-Based Sorting of ELV Plastic Materials

**DOI:** 10.3390/s17061325

**Published:** 2017-06-08

**Authors:** Jiu Huang, Chuyuan Tian, Jingwei Ren, Zhengfu Bian

**Affiliations:** School of Environment Science and Spatial Informatics, China University of Mining and Technology, Xuzhou 221000, China; 07143015@cumt.edu.cn (C.T.); 07143014@cumt.edu.cn (J.R.); zfbian@cumt.edu.cn (Z.B.)

**Keywords:** sensor-based sorting, impact acoustics, ELV recycling, automobile shredder residue

## Abstract

This paper concentrates on a study of a novel multi-sensor aided method by using acoustic and visual sensors for detection, recognition and separation of End-of Life vehicles’ (ELVs) plastic materials, in order to optimize the recycling rate of automotive shredder residues (ASRs). Sensor-based sorting technologies have been utilized for material recycling for the last two decades. One of the problems still remaining results from black and dark dyed plastics which are very difficult to recognize using visual sensors. In this paper a new multi-sensor technology for black plastic recognition and sorting by using impact resonant acoustic emissions (AEs) and laser triangulation scanning was introduced. A pilot sorting system which consists of a 3-dimensional visual sensor and an acoustic sensor was also established; two kinds commonly used vehicle plastics, polypropylene (PP) and acrylonitrile-butadiene-styrene (ABS) and two kinds of modified vehicle plastics, polypropylene/ethylene-propylene-diene-monomer (PP-EPDM) and acrylonitrile-butadiene-styrene/polycarbonate (ABS-PC) were tested. In this study the geometrical features of tested plastic scraps were measured by the visual sensor, and their corresponding impact acoustic emission (AE) signals were acquired by the acoustic sensor. The signal processing and feature extraction of visual data as well as acoustic signals were realized by virtual instruments. Impact acoustic features were recognized by using FFT based power spectral density analysis. The results shows that the characteristics of the tested PP and ABS plastics were totally different, but similar to their respective modified materials. The probability of scrap material recognition rate, i.e., the theoretical sorting efficiency between PP and PP-EPDM, could reach about 50%, and between ABS and ABS-PC it could reach about 75% with diameters ranging from 14 mm to 23 mm, and with exclusion of abnormal impacts, the actual separation rates were 39.2% for PP, 41.4% for PP/EPDM scraps as well as 62.4% for ABS, and 70.8% for ABS/PC scraps. Within the diameter range of 8-13 mm, only 25% of PP and 27% of PP/EPDM scraps, as well as 43% of ABS, and 47% of ABS/PC scraps were finally separated. This research proposes a new approach for sensor-aided automatic recognition and sorting of black plastic materials, it is an effective method for ASR reduction and recycling.

## 1. Introduction

Worldwide the automotive industry has become one of the largest and most developed industries over the last two decades. Nowadays both vehicle production and ownership have reached extremely high levels around the world. The Economic Cooperation and Development Organization (OECD) has estimated that the vehicle growth from the 1990s to 2020s could reach more than 30% [1,2,3,4]. In China in particular, the automotive industry has maintained an annual average growth rate of about 20–25% for the last ten years. In 2014 the total vehicle production reached about 24 million, which made China the largest automotive producer and consumer in the world [4,5,6]. The boom of vehicle ownership has led to a tremendous consumption of fuel and raw materials. Land transport contributes about 20% of the global CO_2_ emissions [2,3,4,5,6,7,8]. Therefore, considering energy conservation and emission reduction purposes, there is a trend to reduce automobile weight by using polymer materials, especially high-performance engineering plastics, instead of metallic materials [7,8,9,10]. Plastic materials can decrease the weight of a whole vehicle by up to 10%, simultaneously reducing fuel consumption by up to 8%. Thus, 100 kg of plastic materials can replace 200–300 kg of metallic materials in vehicles [3,9]. Currently, more and more vehicle parts like dashboards, inner trim, bumpers, fluid tanks, etc. are made of plastics. For example in an Audi-A2 the total amount of plastic materials amounts to 220 kg, which is 24.6% of the vehicle mass [9,10]. Furthermore, the automotive industry has extended the life of vehicles and enhanced End-of-Life vehicles’ (ELVs) generation. In European Union (EU), the average life of vehicles is 10–15 years. In China vehicles are commonly abandoned after 8–10 years. At present, about 15 million ELVs are abandoned annually in the EU, and China is estimated to reach this amount by 2020 [2,5,7,9]. Extensive use of plastic materials and the huge amount of ELVs have created great problems for the recycling, recovery and disposal of ELVs [7,9,10,11,12,13,14,15]. In ELV treatment plants the ELVs get crushed, classified and sorted. Most metallic parts and materials are recycled. Some countries have made significant progress on recycling and recovery of ELVs [14,15,16,17]. For example in the EU, about 60–75% (by mass) of ELVs are recycled as secondary materials. However, this still leaves 25–40% of ELV scraps, which are generally defined as automobile shredder residues (ASRs) or car fluffs [2,3,15,16]. ASR is a solid mixture with extremely high heterogeneity which contains most of the plastics and elastomers from ELVs, in which plastics could account for more than 40% (by mass) [3,7,9]. Due to the high concentration of polymers, ASRs are classified as hazardous materials [1,2,3,8,18,19,20,21,22]. The EU is estimated to currently produce about 2.4 Mt of ASRs annually. ASRs contain lots of pollutants such like heavy metals and chlorine. Hence ASR pyrolysis, chemical and incineration recovery processes could bring new risks and problems to the environment in the form of exhaust gas, ash and residue disposal [21,22,23,24,25,26]. Therefore the main vehicle production countries have implemented legislation for the material recycling and disposal of ELVs and ASRs. The EU Directives 2000/532/EC, 2001/18/EC2, 2001/119/EC3, and 2001/573/EC4 stipulate that from the beginning of January 2015, the ELVs should achieve a comprehensive recovery rate of 95% (by mass), among which the material recycling and parts reuse rate must reach 85% (by mass) within the EU countries [2,3,14,26,27]. In China, the National Development and Reform Commission Proclamation 2006/9 also stipulates that the ELV recovery rate needs to reach at least 95% by 2017, among which material recycling/reuse needs to account for at least 85% [2,3,8,9].

ASR mixtures represent 25–40% by mass of crushed ELVs, in which the fraction of plastic materials is 40–50%, and this proportion keeps increasing. Therefore, in order to fulfill the corresponding directives, novel post-shredder technologies (PSTs) for advanced plastic material recycling from ELVs as secondary raw materials are urgently required, especially for recycling of high value engineering plastics. There are two kinds of ASR sorting and recycling methods: direct and indirect methods. Direct processes like magnetic sorting, eddy current sorting, sink/flotation sorting, etc. utilize corresponding force fields between material scraps and separators. With direct sorting methods ASR mixtures get roughly separated into ferrous metals, nonferrous metals, plastic mixtures, elastomer mixtures, woods and foams and minerals fractions [3,11,13,14,15,16,17]. Further fine sorting of specific fractions to produce pure plastic and elastomer products is still unavailable, since there is currently no effective force field which reacts according to the kind of polymer. 

Indirect sorting processes are another kind of sorting method which concentrates on measureable and identifiable features of individual scraps like their colour, form, texture and visual spectral features. An additional force field like compressed air jets are used for separation power, since there is no adequate force field by which the corresponding particles can be sorted from a mixture stream [2,14,18]. 

In indirect sorting processes, normally the feature extraction, recognition, and scrap sorting are realized using sensors and actuators. Hence this kind of sorting method has been defined as a "sensor- aided sorting" method, which has been developed for more than ten years and revolutionized the design of indirect separation processes [2,9,14]. For example, by using of visual sensors, glass pieces get sorted according to their colours; near infrared (NIR) spectral sensors to recognize and separate different sorts of plastics are well developed. Sensor-based sorting allows the design of systems to achieve recycling efficiencies of 10 t/h [14], but for plastic recycling from ELV the sorting of black and dark dyed plastics is still a problem, since black and dark dyed objects absorb nearly all NIR emissions and as a result produce no reflection. Also painted exterior plastic trim parts can cause recognition errors with NIR. Furthermore, with the development of material science more and more vehicle plastics are being modified with additives, or alloyed with other kinds of plastics. Such modification also causes confusion in NIR spectral analysis [2,14]. Moreover, vehicle plastic modification formulations are always classified as confidential business information, which makes it very difficult for researchers to obtain the data needed to design suitable systems so novel methods that do not rely on NIR spectral data for recognition of black and dark dyed plastics are required for ELV recycling. Recognition using impact acoustic emission (AE) could be an appropriate method. References [28,29,30,31,32,33,34] have reported that this method has been successfully utilized for separation of empty and damaged nuts with high efficiencies of up to 40 pieces/s [29], and this method could find tiny structural failures in rotary facilities and machine parts with high accuracy [31,32,33,34]. Impact AE signals are easily captured and can be processed rapidly by universal computer hardware. For a scrap of a given material, its impact AE features depend only on its geometric characteristics like its shape and particle size [28]. 

Impact AE signals are generated by energy transfer during an impact process and propagate to the ambient in all directions in air medium as longitudinal waves. AE signals can be easily acquired by acoustic sensors like pick-ups and microphones, but in order to recognize plastic materials, acoustic sensors alone are inadequate, since the geometric parameters are also absolutely essential. Therefore for this research a novel visual-acoustic sensor combined detection and recognition method has been developed, in which a 3D visual sensor is used for geometric data detection of plastic scraps, together with an acoustic sensor for acquisition of scrap impact AE signals, as well as the corresponding feature extraction and recognition algorithms have also been designed and combined. 

## 2. Materials and Methods

### 2.1. ASR Plastic Materials Used for This Research

Two kinds of ELV plastics—polypropylene (PP) and acrylonitrile-butadiene-styrene (ABS) scraps—and two kinds of their modified plastics—polypropylene/ethylene-propylene-diene-monomer (PP/EPDM) and acrylonitrile-butadiene-styrene/polycarbonate (ABS/PC) scraps—were sampled directly from an ELV dismantling and recycling plant, in which the samples had been crushed by a shredder. 

PP/EPDM is actually a kind of thermoplastic elastomer (TPE) material, but due to its mechanical properties it performs like a plastic, therefore in this research the PP/EPDM was also considered as a kind of vehicle plastic material. These four kinds of plastics have high temperature variation UV-radiation and oxidation resistance performance, i.e., their mechanical properties are stable in the environment. The four kinds of plastic scrap samples had particle size distributions from 20–400 mm, which were too large for our experimental research, therefore they were further crushed by a cutting mill in our lab using a motor power of 30 kW under 1000 r/min with a screen diameter of 23 mm. 

**Figure 1:**
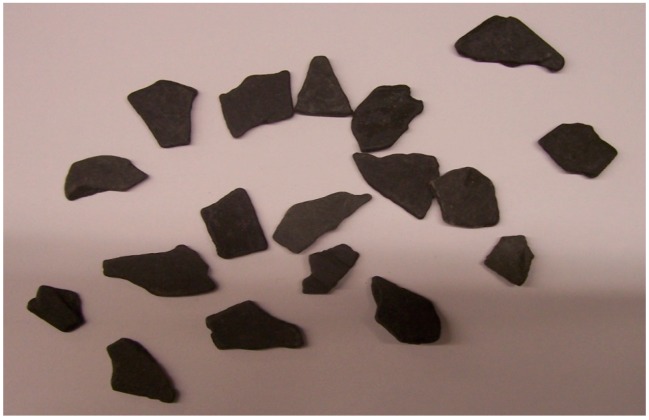
Prepared ELV plastic samples.

The prepared scrap samples have very similar appearance, and the purities of the four kinds of samples were considered as 100%, since they were directly collected from ELVs. The prepared scrap samples are shown in Figure 1, where it can be seen that they all look the same. Almost all the crushed ELV plastic scraps had a flake-like structure, and only a few pieces had cylindrical or other shapes, hence we could consider that all of the scraps have a flake structure. 

### 2.2. Acoustic Emissions during Impact Process

**Figure 2:**
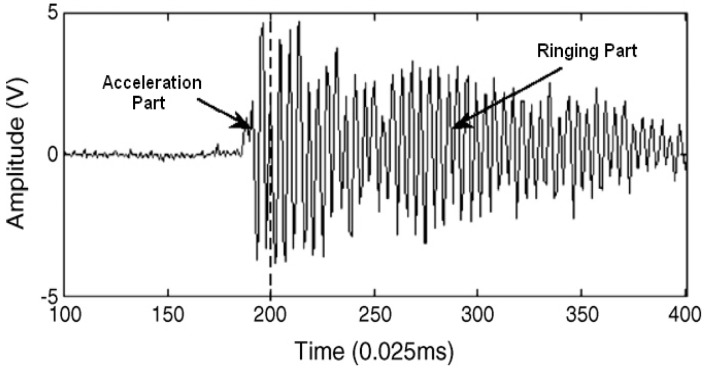
Acceleration and ringing part of an impact AE signal.

The AE induced by impact can be separated into two parts. The first is “acceleration” AE arising from the rapid velocity change of two impacting bodies throughout the impact duration. The second one is the “resonant ringing” AE which is induced by free-vibration energy after the decay of the acceleration energy transfer process [2,28]. Resonant ringing AE is the impact frequency response after the decay of the acceleration AE, which is the natural frequency of the impacted body and traditionally recognized as a feature for integrity recognition of materials or structures. This method has been widely reported in the literature. Acceleration sounds and resonant ringing sounds are shown in Figure 2 [28].

### 2.3. Impact Acoustic Emission (AE) Response of Flake Structure Scraps

Most vehicle plastic parts are produced with hollow and shell structures, such as frames, dashboards, tanks, bumpers, etc. Therefore after crushing, most plastic scraps have flake-like structure shapes, which could be considered as 2-dimensional flat shapes with corresponding thicknesses. The impact between ASR plastic scraps and another rigid body can be considered as an elastic collision, which means that before and after impact, there are neither permanent deformations nor ruptures on the impact bodies, i.e., all of the kinetic energy loss has been transferred to be vibration of both impact bodies and then propagates to the ambient air.

A light impact velocity produces and elastic deformation in both impact bodies. For flake structures, it can be defined that the vibration energy loss factor *λ* is the ratio of the energy transferred into flake free-vibration *E_f_* of the flake scrap to the overall energy *Esum*, which can be written as [28]:

(1) $$\lambda =\frac{E_{f}}{E_{sum}}=\frac{1}{16}\sqrt{\frac{M_{p}\;K_{c}}{\rho \;h\;K_{f}}}$$

where *h* and *ρ* are the thickness and density of flake scraps, respectively, *M_p_* represents the effective mass of scraps, *K_f_* and *K_c_* represent the bending stiffness and non-linear contact stiffness of scraps, then we get:

(2) $$K_{c}=\frac{4}{3}E\sqrt{R}$$

(3) $$K_{f}=\frac{4\pi h^{3}}{3b^{2}}(\frac{E_{2}}{1-v_{2}^{2}})$$

where *b* is the radius of the flake scraps, and parameters *R* and *E* are defined as:

(4) $$\frac{1}{R}=\frac{1}{R_{1}}+\frac{1}{R_{2}}$$

(5) $$\frac{1}{E}=\frac{1-v_{1}^{2}}{E_{1}}+\frac{1-v_{2}^{2}}{E_{2}}$$

Herein *R*_1_ and *R*_2_ are the equivalent radius of the two impacting bodies (for this case of the impact between plastic scraps and impact plate). The Young’s modulus and Poisson’s ratios of the plastic scraps and impact plate are *E*_1_, *v*_1_ and *E*_2_, *v*_1_, respectively. Setting:

(6) $$k_{12}=\frac{\frac{1-v_{1}^{2}}{E_{1}}}{\frac{1-v_{2}^{2}}{E_{2}}}$$

and *R*_1_ = *a*, considering $M_{p}=\rho \pi ha^{2}$, λ can be obtained as:

(7) $$\lambda \approx \frac{1}{16}\frac{\sqrt[{4}]{a}}{\sqrt{1+k_{12}}}\frac{b^{2}}{h\sqrt{h}}$$

Therefore, for a scrap/flake structure with the same radius *b*, a small *h* would cause a high *λ*, which means a strong initial acceleration. It is thus seen that for thin flakes, heavy acceleration sounds will be radiated by the large initial bending acceleration of the flake structures. On the other hand, with a big *h* a small λ results, and few acceleration sounds will be generated, hence a thick and heavy stone plate was chosen to represent a rigid impact plate.

For convenience, the crushed plastic scraps were equivalently considered as a circular flakes with different equivalent diameters. According to vibration principles, the propagation equation for impact pressure waves in a uniform thin flake along the transverse axis *z* is [30]:

(8) $$\frac{\partial^{2}z}{\partial t^{2}}+\frac{Eh^{2}}{12\rho (1-v^{2})}\nabla^{4}z=0$$

where *E* is the Young’s modulus, ρ is the material density,*v* is the Poisson’s ratio of the plate material, and *h* is the scrap thickness. 

For harmonic solutions, *z = Z*(*x, y*)*e^j^^ω^^t^*, where ω is the angular flexural vibration frequency:

(9) $$\nabla^{4}Z-\frac{12\rho (1-v^{2})\omega^{2}}{Eh^{2}}Z=\nabla^{4}Z-k^{4}Z=0$$

where:

(10) $$k^{2}=\frac{\sqrt{12\;}\omega }{h}\sqrt{\frac{\rho \;(1-v^{2})}{E}}=\frac{\sqrt{12}\omega }{c_{p}h}$$

*C_p_* is the velocity of the pressure wave in an infinite flake structure. In the case of a circular plate of radius *b* and thickness *h*, with fixed boundary, the frequency response of impact AE is determined by the equation:

(11) $$f_{m,n}=\alpha_{m,n}\sqrt{\frac{E}{\rho \;(1-v^{2})}}\frac{h}{b^{2}}$$

where *m* and *n* are integers (beginning with zero), *α_m,n_* is a dimensionless coefficient associated with the corresponding flexural vibration mode (*m*,*n*), which depends on different modes of impacts, i.e., the impact position and shape of impact bodies.

According to Equation (11), the material property parameters of*ρ*, *E*, *b*, *h* are known, and *f* can be obtained from the impact AE signals. The Poisson’s ratio *v* is a dimensionless quantity that depends only on material. It can be deduced that the unit of coefficient*α_m,n_* should be mm/s or m/s, which are the units of velocity.

From Equation (11), the coefficient*α_m,n_* could be further determined as a dimensionless constant quantity, since the impact processes always occurs on the edge of scraps. Hence in this research, the impact frequency response for a specific material depends only on the thickness and radius of single scraps. Combining the operation of all constants as *k*:

(12) $$f=k\frac{h}{b^{2}},\;(k=\alpha_{m,n}\sqrt{\frac{E}{\rho (1-v^{2})}})$$

Obviously *k* is also a constant for a given material. Here the impact frequency response could be simplified as a function of scrap particle size and thickness. Equation (12) could be further normalized by using a standard thickness *h_norm_* = 1 mm and the particle size *D* instead of radius *b* as: $f_{norm}=\frac{f}{4h}=k\frac{1}{D^{2}}$ and further:

(13) $$f_{norm}=k_{C}\frac{h_{norm}}{D^{2}},\;(h_{norm}=1\;mm)$$

The coefficient *k_C_* has units of mm/s or m/s, the same as the units of velocity, which indicates the variation of different plastic materials. Equation (13) also shows that for scraps of a given plastic material, *k_C_* is constant and the impact frequency response depends only on the thickness and particle size of a scrap. The value of *k_C_* varies according to the plastic material considered. With the help of Equation (13) we could avoid direct measurements of plastics’ property parameters like Young’s modulus, density, Poisson’s ratio, etc. Different kinds of materials could be identified by determination of *k_C_* by using fitting and regression analysis of impact frequency responses.

### 2.4. Determination of the Flake Equivalent Diameter by Fine Screening

The particle sizes of crushed scrap were formed randomly during the shredding process. Generally all of them were maintained a flake-like structure. According to Equation (13) the scraps were considered as circular shape flakes with equivalent diameters. Fine screening could be an appropriate method for the determination of equivalent diameters. In this research all of the sampled ELV plastic scraps were further crushed with a cutting mill and then fine sieved with mm sieving fractions from 8 to 23 mm. The minimum screen aperture, through which the corresponding particles pass determines the particle size/equivalent diameter of scraps. For example, if a certain number of scraps could pass 9 mm screen but they are rejected by a 8 mm screen, it can be determined that the the equivalent diameter of this amount of these scraps is 9 mm. 

### 2.5. Determination of the Flake Thickness by Using 3D Laser Triangulation

3D laser triangulation is an algorithm used to measure the range/position/thickness of an object using a CCD sensor according to the triangular relationships between the laser emitter, camera, and object. Single or multiple non-scattering and monochrome laser-beam emitters are commonly used in triangulation methods [35,36,37]. 

**Figure 3:**
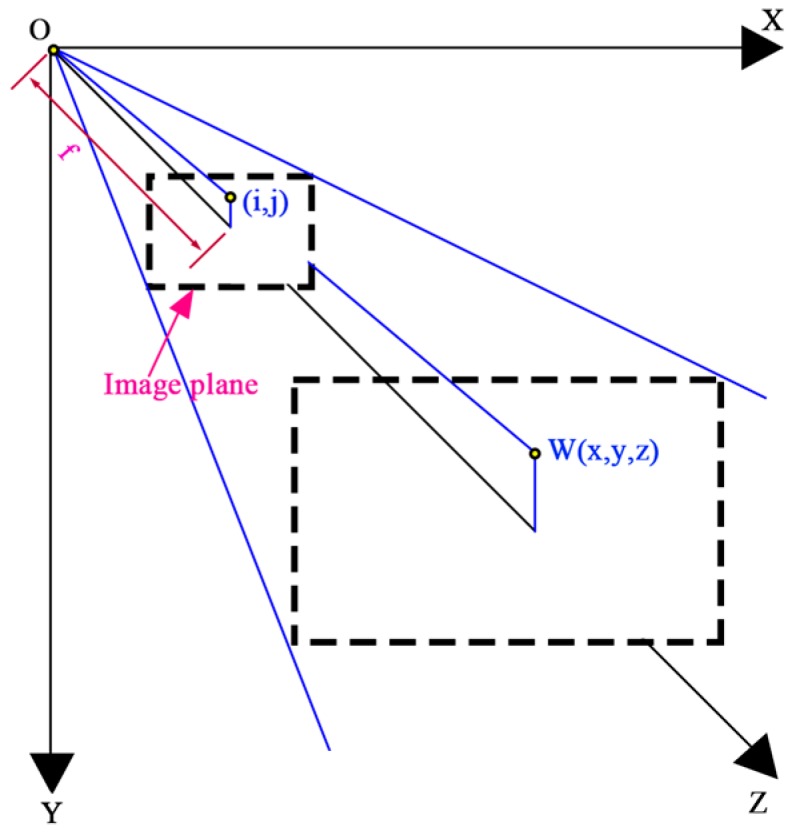
3D Laser triangulation scanning measurement.

**Figure 4:**
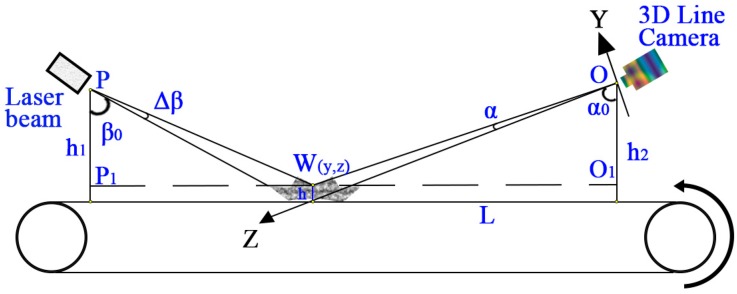
CCD camera imaging.

Modern industrial digital line scan cameras are ideal visual sensor for laser triangulation. For example, some line scan camera products measure the 3D visual parameters, positions and color of objects at the same time with line scan frequencies of up to several kHz and achieve a resolution of more than 1 Megapixel. In this study the tested plastic scraps were dropped individually and transferred on a conveyor belt. One laser beam was adequate for the 3D scans. The laser beam emitter had a monochrome wavelength about 660 nm and the laser beam covered the width of the conveyor belt in the vertical direction. The installation for 3D measurement and CCD sensor imaging through a camera are shown in Figure 3 and Figure 4 [36,38]. 

As shown in Figure 3 and Figure 4, the camera is installed along the Z axis with the focal point of the lens located at the origin point of the XYZ coordinate system. As shown in Figure 3, depending on how far away the laser strikes a surface, the laser beam images different grayscales on the CCD sensor area of view. This is the principle of laser triangulation scanning because the laser beam, the camera and the laser emitter form a triangle. The focal point of the camera and the laser beam emitting point were *O* and *P*, respectively. The laser emitter emits a laser beam onto the conveyor belt surface. According to the belt surface, the heights of the camera and the laser emitter are *h*_1_ and *h*_2_, and the height/depth of the measured surface point *W* is *h*. *L* is the horizontal distance between the camera and the laser emitter, and $L=\vec{O_{1}P_{1}}$. *α*_0_ is the installation angle of the lens axis in the vertical direction, and α is the angle between line $\vec{OW}\;$ and the lens axis. *β*_0_ is the setting angle of laser beam to the vertical direction, and *Δβ* is the incremental beam angle of laser line. From triangles *ΔOO*_1_*W* and *ΔPP*_1_*W*, the following equation can be obtained [36]:

(14) $$\tan(\alpha +\alpha_{0})=\frac{L-(h_{1}-h)\tan(\beta +\Delta \beta )}{h_{2}-h}$$

and:

(15) $$\tan\alpha =\frac{y}{z}$$

If *f* is the focal length of the camera and point (*i*, *j*) is the pixel position in the image area of CCD sensor that corresponds to a point *W(x*,*y*,*z)* on the object surface, then two similar triangle relationships can be determined as:

(16) $$\frac{y}{j}=\frac{z}{f}$$

Further the measurement of scrap thickness *h* could be written as:

(17) $$h=\frac{[L-h_{1}\tan(\beta +\Delta \beta )](f-j\tan\alpha_{0})-(j-f\tan\alpha_{0})h_{2}}{(f-j\tan\alpha_{0})\tan(\beta +\Delta \beta )+f\tan\alpha_{0}+j}$$

For Equation (17), *j* is the only variable which need to be determined from the acquired images. Others are fixed parameters of the facility installation. In this study, there was one laser beam emitted vertically onto the belt surface, hence the angles *β*_0_ and *Δβ* were zero. Therefore Equation (17) can be rewritten as:

(18) $$h=\frac{L\;(f-j\tan\alpha_{0})-(j-f\tan\alpha_{0})\;h_{2}}{f\tan\alpha_{0}+j}$$

**Figure 5:**
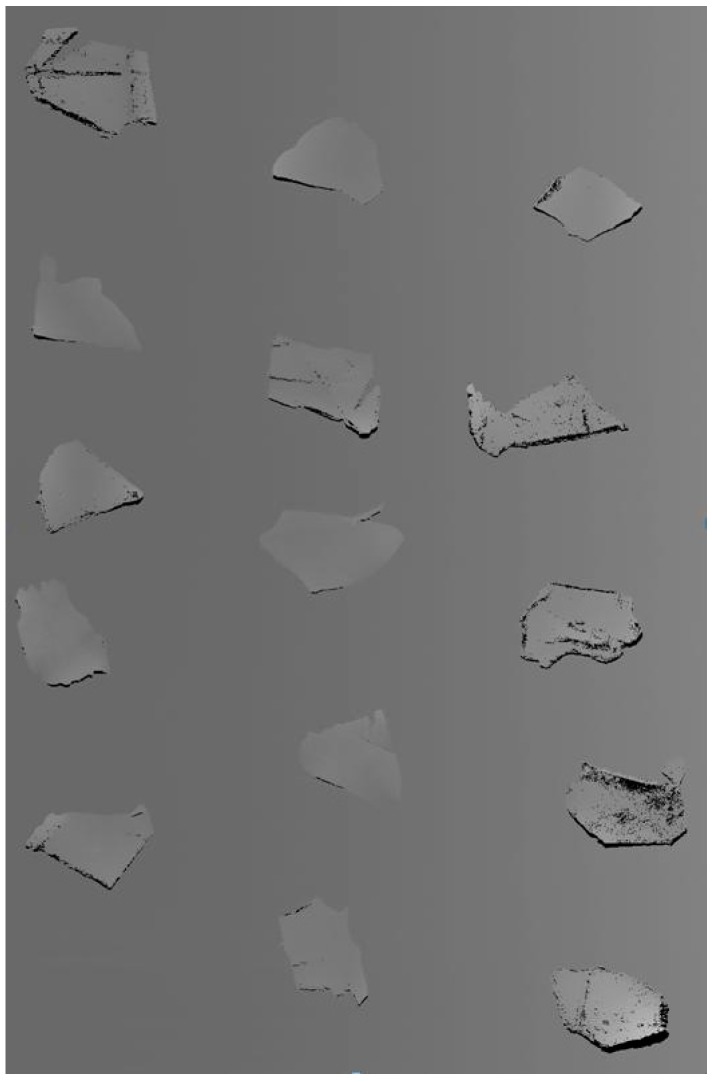
3D grayscale image.

The tested scraps were scanned and imaged as grayscale images, and example of which is shown in Figure 5, which shows that the thicknesses of tested scraps with different thickness were observed as different grayscales.

**Figure 6:**
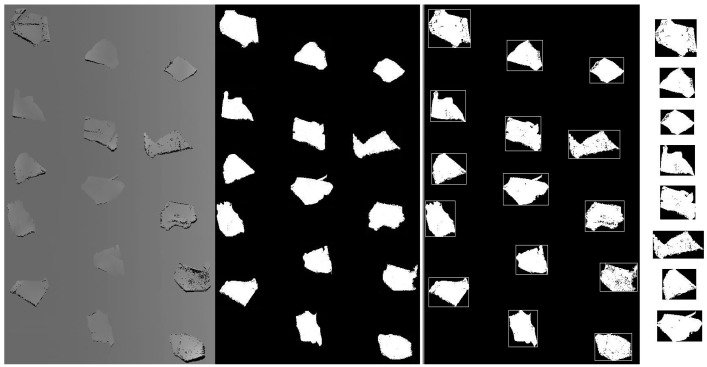
Image segmentation using a binary mask and AABB.

Then all of the scrap images were segmented by using binary grayscale image and axial-aligned-bounding-box (AABB) masks. This processes is shown in Figure 6.

As shown in Figure 6, the 3D grayscale images are converted to binary images firstly according to:

(19) $$o_{ij}=\{\begin{array}{c} 1\;(o_{ij}>g*)\\ 0\;(o_{ij}<g*) \end{array}$$

where *o_ij_* is the grayscale value at the image pixel x = *i*, y = *j*. The grayscale value 1 means the pixel is 100% white and the gray scale value 0 means the pixel is 100% black, g* is the threshold grayscale value. 

On binary images, the position information of tested scraps could be navigated and then segmented by using an edge detection algorithm like the Prewitt or Sobel operators, which could find edges of objects by finding the biggest changes of the grayscale intensity. After the detection of scrap edges, the 2D axial-aligned-bounding-boxes (AABB) algorithm was used to segment the scrap masks by using the maximum pixel value of the binary mask in the ±x and ±y directions. Than we relocated the segmented masks of each scrap image back to the original grayscale image, where the position information and grayscale of each scrap could be segmented and further the thickness of each scrap was calculated. 

## 3. Experimental Setup

### 3.1. Implemented Acoustic and Visual Sensor

**Figure 7:**
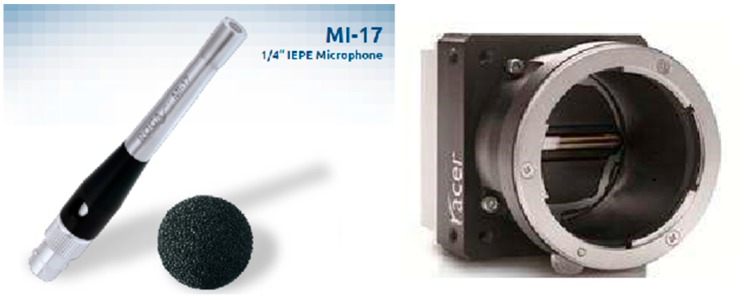
Implemented acoustic and visual sensors.

An industrial ROGA MI-17 ICP acoustic pick-up was used for impact AE acquisition. Its frequency response covers the 20–22 kHz range with a sensitivity about 50 mV/Pa. The AE signal preprocessing device was a 16 bit computer soundcard, whose performance is adequate for industrial measurement at a much cheaper price than professional DAQ devices. In computers the soundcards transfer data by a Direct Memory Access (DMA) algorithm and this results in a massive reduction of CPU use. Meanwhile the computer Peripheral Component Interconnect (PCI) bus allows high speed data communication between the soundcard and the system which made the online measurement, real time analysis, as well as real time manipulation by using virtual instruments feasible. The visual sensor was a *BASLER AT C3-1280-CL* line scan camera with a highest scan frequency of 47000 lines/s and a resolution of 1280 pixels. The laser beam for 3D measuring had a wavelength of 660 nm. The acoustic and visual sensors are shown in Figure 7.

### 3.2. Acquisition Facility of Impact Acoustic Emissions

**Figure 8:**
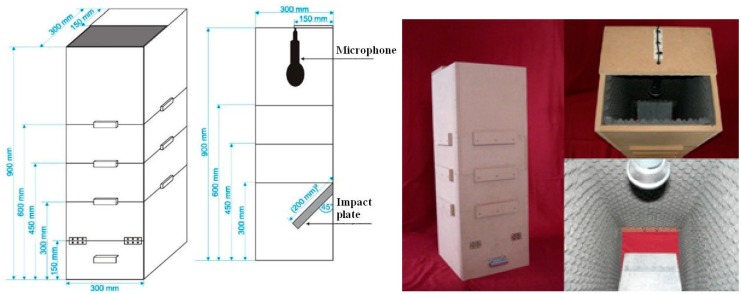
Impact AE signal acquisition facility.

In order to acquire impact AE signals, plastic scraps were designed to be dropped from a defined height from conveyor belt and then impacted one by one on a thick stone plate. The impact test process was sealed in an empty medium density fiberboard (MDF) case whose inner surface was covered by sponge material in order to isolate ambient noises. The MDF chamber consisted of four elements. The height of the case can be adjusted to be 600, 750 or 900 mm for different falling heights. The outer dimensions of this equipment were 300 mm in width and 300 mm in length, and the thickness of the MDF plates was 15 mm. The inner dimensions were about 270 mm in width and 270 mm in length. The impactor was a stone plate which was made of nature stone for foot paths with dimensions of 200 mm × 200 mm × 30 mm. The stone plate was placed in a steel bracket and set at a 45° angle inside the system. The acoustic signals of impacts were acquired by the acoustic pick-up which was placed at the top of the inner space. This chamber is shown in Figure 8. The reason for the selection of a heavy stone was the excellent stability of the stone plate and its impact vibration characteristics which are very different from those of the polymers. 

### 3.3. Setups of Impact AE Acquisition 

According to the Nyquist-Shannon sampling theorem, the sampling rate of the acoustic pick-up was set to be 44,100 Hz, and the sampling period was set to be 0.1 s. The window function for signal segmentation was set to be a Hanning window, since it generated relatively lower side lobes in the frequency spectrum i.e., lower signal energy leakage. The 3D triangulation scanning images were set to be 32 bit grayscale, in order to increase the scrap thickness resolution. 

### 3.4. Installation of the Whole System

**Figure 9:**
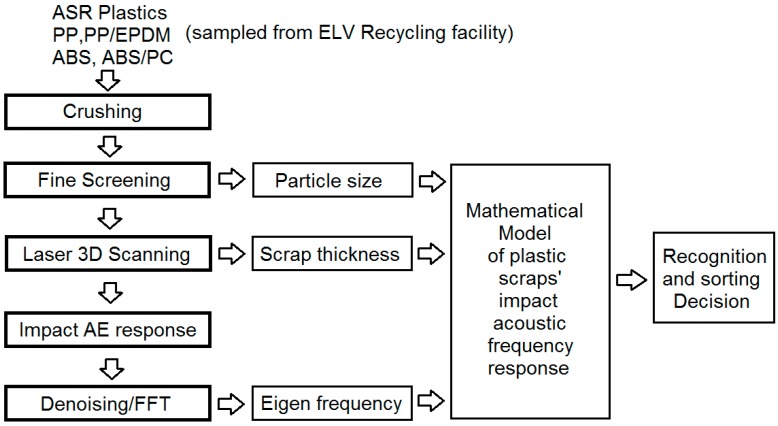
Recognition and sorting processes of ELV plastic scraps.

Generally the whole experimental setup of this research consists five practical processes and three data evaluation/determination processes, which are shown in Figure 9.

During this research, the plastic scraps were dropped one by one individually by a vibration feeder onto a conveyor belt on which their thicknesses were measured. At the end of the conveyor belt the plastic scraps fell into the impact chamber freely whereupon the impact processes occurred and the AE signals were captured. Two optical triggers were fitted at the entrance and exit of the chamber, respectively, by which the acoustic pick-up was triggered to acquire the impact AE of the impacted scraps sequentially according to the thickness data of the impacted scraps; and the compressed air nozzle array was also triggered together with a computer in order to blow the recognized scraps out of the main material stream and get sorted. Reference [29] mentioned that the nozzle array separation frequency could achieve theoretically 40 pieces per second, but in this research, in order to insure the exact acquisition of each impact, we extended the spacing time between two impacts, and the dropping of scraps was set to be about 1–2 pieces/s. Certainly this velocity could be increased once this research has obtained robust results and manipulation parameters.

**Figure 10:**
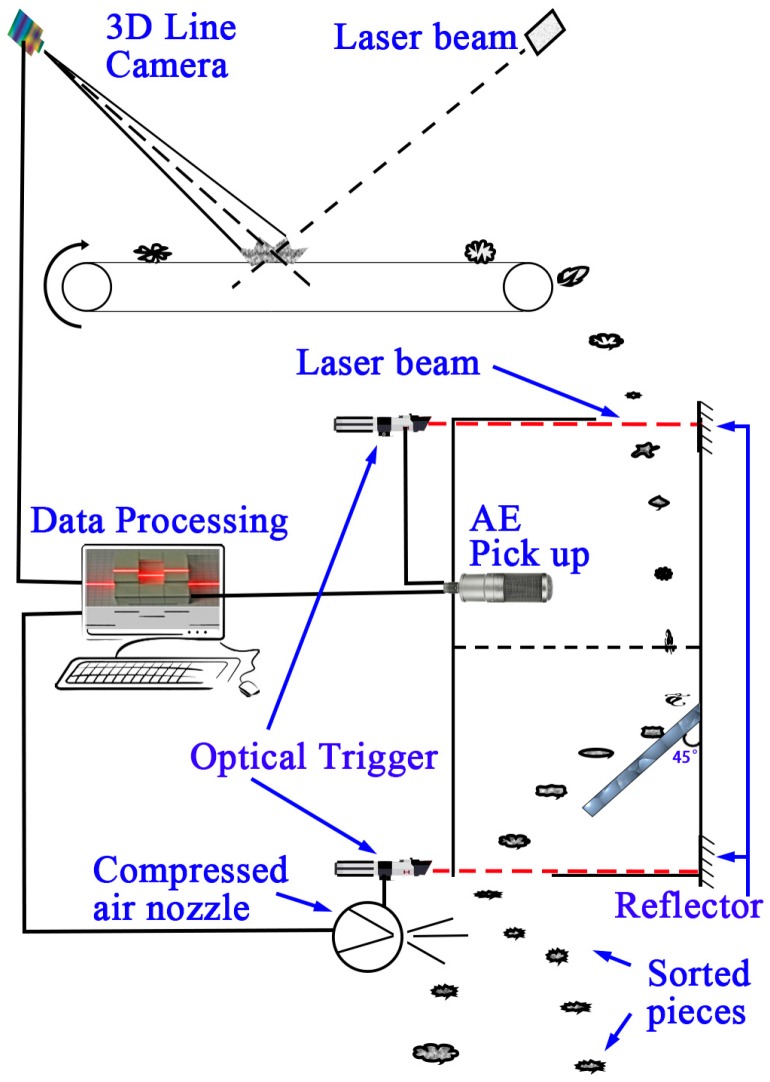
The experimental system.

The installation of the experimental system is illustrated in Figure 10, where the impact AE acquisition chamber was installed under the end of the conveyor belt, which let the tested scraps fall into the chamber, and the visual sensor and laser beam were placed on the frame structures above the conveyor belt.

## 4. Results and Discussion

### 4.1. Impact AE Signal Pre-Processing and Filtration

**Figure 11:**
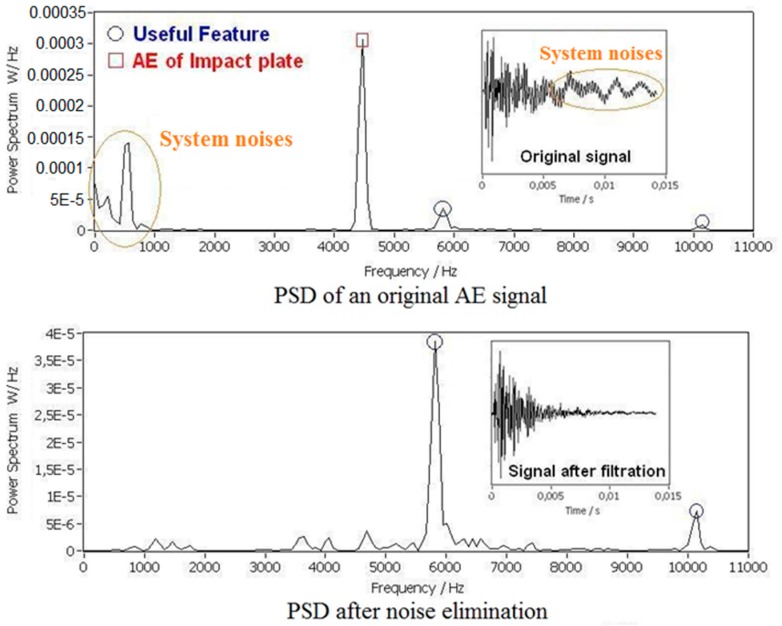
PSD of impact AE signal before and after filtration.

In this study all the impact AE signals were captured in form of 440–500 samples with 8 bit within 10–100 ms. The captured AE signals were processed and analyzed by the power spectral density (PSD) method and wavelet transformation in the frequency domain. In the time domain the features of the AE signals were difficult to recognize. The main features of AE signals were the impact resonant ringing energy distribution in the frequency domain. The power spectral density has better contrast than the impact resonant energy distribution as illustrated by the peaks and valleys located in the corresponding frequency domain of the power spectral density [32,33]. Acquired impact AE signals contained significant noises, such as the AE signals of the stone plate impactor and the system white noises which were generated by the electronic devices. The noises were filtered by digital filters. An example of the AE signal filtration of a PP scrap piece is shown in Figure 11.

**Figure 12:**
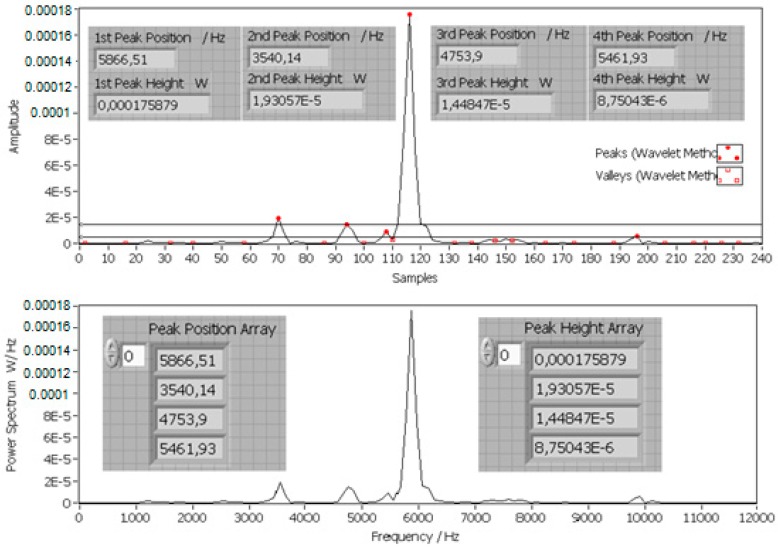
Feature extraction from the power spectrum.

The features of the impact AE were extracted by peaks and valleys recognition through a wavelet transform, which is shown in Figure 12. Both the positions and heights of peaks and valleys were recognized and rearranged to be feature arrays. Only the peaks and valleys with heights larger than a threshold were considered as features.

For the impact AE signals in this research, the positions and heights of the first peak were the most important features according to the mathematical model of the impact AE resonant frequency responses shown in Equation (13). Although impact scraps were approximately considered to be circular, the main peak positions occurred in a narrow domain around the feature peak position that corresponded to their equivalent diameter in the power spectral density. Other peaks could be also generated by irregularities on the scrap surface, for example nicks and fractures, but the intensity of these peaks was much lower than that of the main feature peaks. 

### 4.2. Determination of Impact Frequency Response Coefficient k_C_

The position of the first peak in the power spectral density analysis was the key feature of the impact AE frequency response. Together with the thicknesses of scraps which were measured by the 3D visual sensor and the equivalent diameters of scraps which were measured by fine screening, the impact frequency response coefficients *k_C_* could be determined by using fitting and regression analysis. According to the model of Equation (13), each material had its own distribution of frequency responses according to its scrap equivalent diameter *D*. By comparing with the computer database the recognition could be realized in 20–40 ms. 

**Figure 13:**
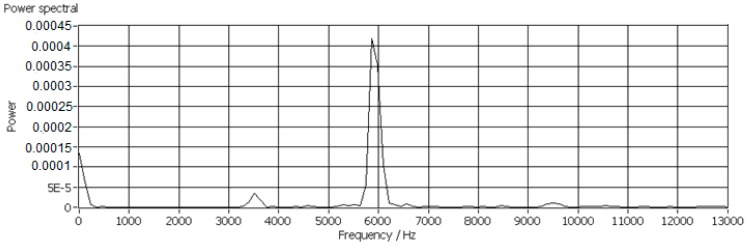
Impact AE frequency response of a PP scrap with an equivalent diameter of 14 mm.

**Figure 14:**
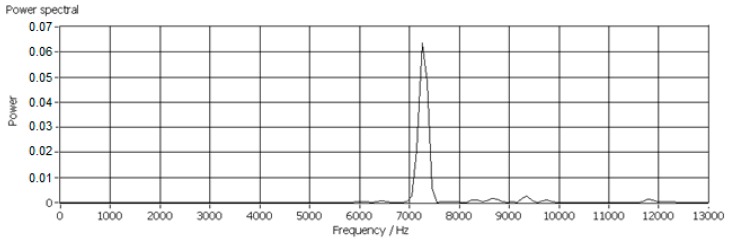
Impact AE frequency response of an ABS scrap with an equivalent diameter of 18 mm.

**Figure 15:**
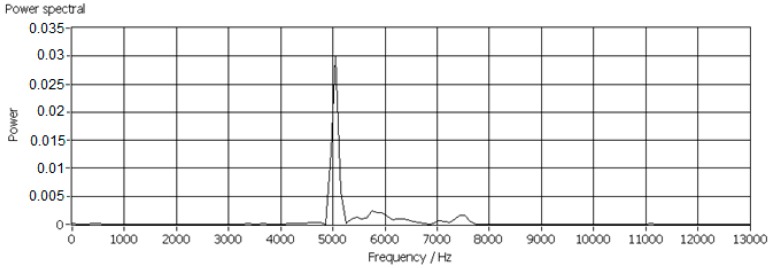
Impact AE frequency response of a PP/EPDM scrap with an equivalent diameter of 14 mm.

In this research work, more than 600 scraps of each material with equivalent diameters ranging from 14 mm to 23 mm were tested for determination of the *k_C_*, since the mass of the scraps with diameters ranging from 14–23 mm was sufficient to generate AE signals with adequate strength. Low mass scraps generated weak AE signals which were usually covered by background noises and the scraps larger than 23 mm were too big for this test facility. Typical power spectra of impact AE frequency responses are shown in Figure 13, Figure 14 and Figure 15.

**Figure 16:**
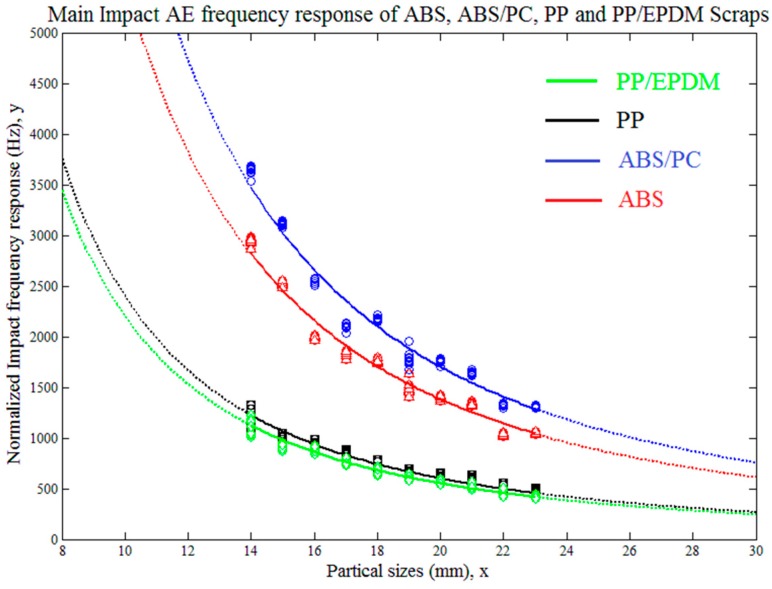
Fitting analysis of the tested materials’ impact AE frequency responses.

The fitting analysis was also set to start from the particle size 14 mm to 23 mm. The fitting and regression model had been theoretically derived in Equation (13). The results of the fitting analysis of the PP, PP/EPDM, ABS and ABS/PC material scraps are shown in Figure 16, respectively.

Figure 16 shows the impact AE frequency response distributions of polypropylene (PP), acrylonitrile-butadiene-styrene (ABS), polypropylene/ethylene-propylene-diene-monomer (PP-EPDM) and acrylonitrile-butadiene-styrene/polycarbonate (ABS-PC) according to the scraps’ equivalent diameter. The impact frequency coefficients *k_C_* of the tested materials were also fitted, and they were:

For PP, the fitted value of *k_C_*_1_ was 240.75 m/s with a fitting coefficient *R*^2^*(PP)* = 0.9238;
For PP/EPDM, the fitted value of *k_C_*_2_ was 220.83 m/s with a fitting coefficient *R*^2^*(PP/EPDM)* = 0.9079;
For ABS, the fitted value of *k_C_*_3_ was 551.4 m/s with a fitting coefficient *R*^2^*(ABS)* = 0.8612;
For ABS/PC, the fitted value of *k_C_*_4_ was 682.2 m/s with a fitting coefficient *R*^2^*(ABS/PC)* = 0.8287.

The values of the coefficient *k_C_* indicated the characteristic and feature variations of the tested materials. Figure 16 shows that the feature distribution between PP-based and ABS-based materials had obvious differences and departed far from each other, which means PP-based plastics and ABS-based plastics could be completely distinguished by using impact frequency response, which means they are theoretically able to be completely sorted. However the coefficients *k_C_*_1_ and *k_C_*_2_ of PP and PP/EPDM were very close, and so were the coefficients *k_C_*_3_ and *k_C_*_4_ of ABS and ABS/PC. Hence the frequency response distribution domains between the tested PP and PP/EPDM scraps, as well as between the tested ABS and ABS/PC scraps overlapped. The overlapping of impact frequency responses indicated that by material modification with EPDM in PP, and PC in ABS, the mechanical properties of the corresponding vehicle plastics reflected only the limited changes and optimization according to the requirements of the vehicle parts. And these two kinds of material modification did not provide adequate differences in the impact acoustic features and this led to limited recognition and separation efficiency. Therefore, further studies on the sorting efficiency between PP and PP/EPDM as well as between ABS and ABS/PC are necessary. 

**Figure 17:**
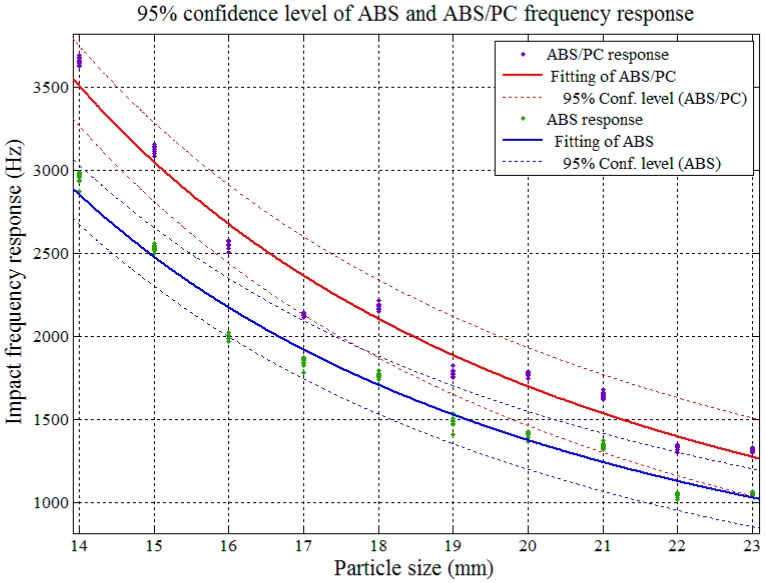
Confidence interval domain of ABS and ABS/PC at the 95% confidence level.

**Figure 18:**
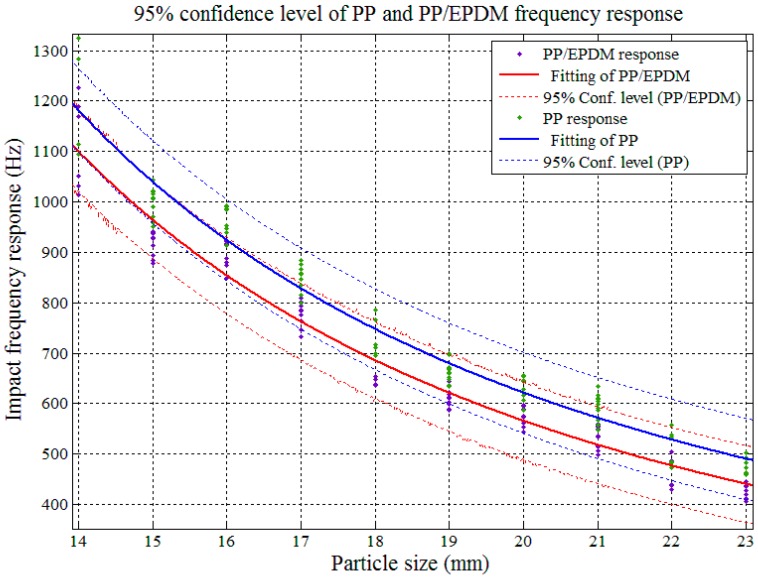
Confidence interval domain of PP and PP/EPDM at the 95% confidence level.

**Figure 19:**
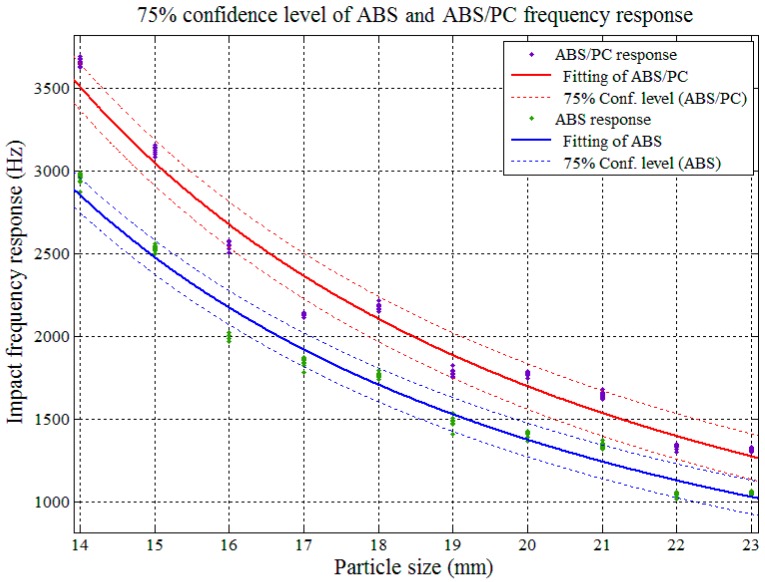
Confidence interval domain of ABS and ABS/PC at the 75% confidence level.

**Figure 20:**
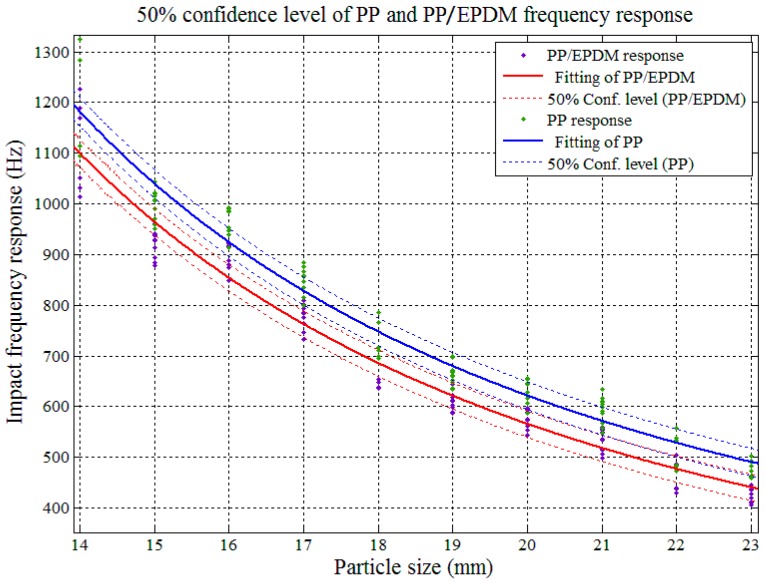
Confidence interval domain of PP and PP/EPDM at the 50% confidence level.

The impact AE frequency responses’ confidence intervals between PP and PP/EPDM as well as ABS and ABS/PC were required in order to determine the theoretical sorting accuracy among pure and modified plastics. The confidence interval analysis and the results are shown in Figure 17, Figure 18, Figure 19 and Figure 20.

Confidence level is a statistical definition which is the frequency (i.e., the proportion) of confidence intervals that contain the true value of the corresponding parameter. In other words, if confidence intervals were constructed using a given confidence level in a huge number of independent experiments, the proportion of those intervals that contained the true value of the parameter could match the confidence level. If we set the original hypothesis as the impact frequency response located in the confidence interval was true, and we might use *α* as the probability (significance level) of original hypothesis rejecting, then the confidence level could be defined as 1 − *α*.

In Figure 17 and Figure 18, both of the impact AE frequency response confidence intervals between PP and PP/EPDM scraps as well as ABS and ABS/PC scraps were overlapped at the 95%confidence level. The overlapping between ABS and ABS/PC started at the particle size of 17.4 mm, and for PP and PP/EPDM the overlapping covered the whole tested particle size range, and expected to be maintained on both sides of the scrap sizes extension. Therefore we need to reduce the confidence level in order to decrease the confidence interval overlap and study the theoretical recognition efficiency and separation rate between PP and PP/EPDM as well as ABS and ABS/PC. 

Figure 19 shows that with a confidence level of 75%, the confidence intervals of the impact AE response for ABS and ABS/PC departed from each other in the particle size range of 14–23 mm, which means in this range, if we use the impact AE response features located in the confidence intervals with 75% confidence level as recognition criterion, the theoretical separation rate could achieve a maximum 75% value. 

Figure 20 shows that with the confidence level set at 50%, the confidence interval of the impact AE responses for PP and PP/EPDM had no overlap in the particle size range of 14–23 mm, which means in this range, if we use the impact AE response features located in the confidence interval with 50% confidence level as recognition criterion, the theoretical separation rate could achieve a maximum 50% value.

The experimental results also demonstrated that as the impacted scrap particle size increased, the fitting curve of the original and modified plastic materials got closer to each other, which could cause confidence interval overlapping and reduce the separation efficiency. With the decreasing of impact scrap particle sizes, the fitting curves departed further from each other, which provided more space to increase the confidence level, which indicated that if we need to obtain higher material recognition and separation rate accuracy, we need to adjust the crushing process of the end-of-life vehicle plastics to have more fractions of a relatively smaller particle size. However, in contrast, smaller scraps generate low intensity AE emissions, which are easily masked by system and ambient noises, and the plastic materials could not be crushed to be extremely small, since the fine crushed particles could not be considered as having a flake structure anymore and in this situation the impact AE frequency response model described by Equation (13) is no longer valid. Hence how to find an appropriate crushed particle size range for ELV plastics is an important theme for future study. 

According to the experimental results, of course we could in the future optimize the recognition efficiency by using highly sensitive sensors and data acquisition devices like professional acoustic signal sensors and data processing cards. However, a more basic solution is to determine the ranges of appropriate particle size for ELV plastics, and we should also study a suitable crushing method, in order to concentrate a higher percentage of the scraps in the appropriate particle size fractions.

The impact AE acquisition chamber used in this research is much smaller than the actual containers used in ELV recycling facilities. Obviously, with higher falling height the tested scraps could have much more impact energy and generate much stronger signals, which could make small scraps be successfully recognized. In the future we could implement the experiment in actual facilities for a broader range of particle sizes. 

### 4.3. Recognition and Sorting Test Based on the Determination of Impact Frequency Response Coefficient k_C_

**Figure 21:**
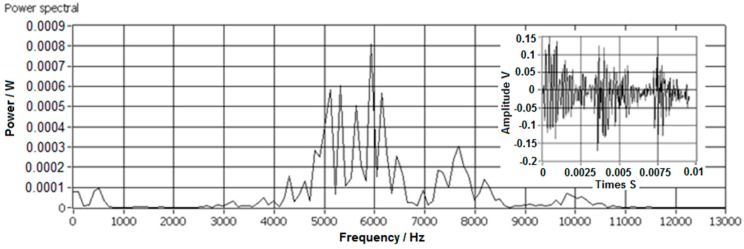
Abnormal impact by triple impaction.

Theoretically, according to the fitting analysis of the coefficient *k_C_* and the confidence interval with corresponding confidence level according to the large amount of actual impact AE frequency responses of each scraps, the recognition and sorting accuracies could be determined. After determination of the impact AE frequency response coefficients *k_C_* in Figure 17, Figure 18, Figure 19 and Figure 20, an actual test was implemented by using the remaining crushed sampled materials from the ELV recycling plant. The results show that the PP-based materials (PP, PP/EPDM mixture) and ABS-based materials (ABS, ABS/PC mixture) were recognized and separated completely except for several abnormal impacts that amounted to less than 1% (by mass), but the actual recognition and sorting accuracies between pure and modified plastics were 39.2% for PP and 41.4% for PP/EPDM scraps, and 62.4% for ABS, and 70.8% for ABS/PC scraps with diameters ranging from 14 mm to 23 mm, respectively, which were much lower than the theoretical estimation. For the diameter range of 8–13 mm, only 25% of PP, and 27% of PP/EPDM scraps as well as 43% of ABS, and 47% of ABS/PC scraps were finally separated. Within the diameter range of less than 8 mm, only a very limited number of scraps were recognized. The reasons of this decrease and recognition failure can be explained as follows:

(1) For the scraps with a diameter range of 14–23 mm, several abnormal impacts caused by double or triple impactions on the stone plate occurred, which led to aliasing of the real frequency response. A typical abnormal impact and its power spectrum are shown in Figure 21.

Since the impact process occurred randomly, the abnormal impacts could not be avoided but we could reduce probability of their occurrence by decreasing the installation angle of the impact stone plate which has been described in Figure 8. By decreasing the angle of the impact stone plate, the effective impact area could be reduced and therefore the probability of multiple impacts reduced. 

**Figure 22:**
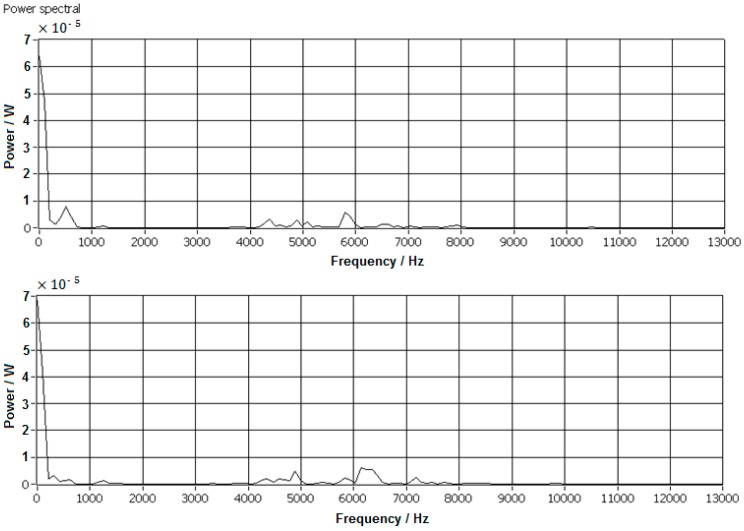
Impacts with low mass scraps.

(2) With decreasing scrap diameter, the scrap mass decreased, hence the impact energy was also reduced, which led to inadequate signal strength for PSD analysis or the signals were hidden by system noises. Typical low energy power spectra are shown in Figure 22.

Figure 22 shows that the impact energy was too low to be detected by the AE acquisition sensor, or the signals were covered by background system noises. All peaks in the power spectra were lower than 10^−5^ W, which is much lower than the system noises. With the decrease of scrap diameter, more and more of the impact AE power spectrum became unapparent, the features are confused with system noises and this leads to incorrect or failure in recognition and separation. When the scraps’ diameter was less than 8 mm, most of the impact AE power spectrum was unable to be recognized. 

Hence, according to the experimental results, the main limitation of this method was verified to be the shape of scraps, as well as the particle sizes. Considering our separation objects, almost all of the ELV plastic scraps had flake shapes, since the corresponding vehicle parts had hollow structures, bur crushed scraps with too small particle size could not be avoided during the crushing process, which was the main restriction of the recognition and separating efficiency. 

Although this method still cannot be applied to considerable parts of the small size fractions, it does provide a new and effective way to reduce the amount of ASR disposal, since it realized complete sorting and material recycling of PP-based and ABS-based ELV plastics, which constitute the largest proportion of vehicle plastics. If we need to further recycle the PP/EPDM and ABS/PC from PP- and ABS-based plastics, respectively, and then directly use the recycled products for new vehicle production, this method also provides a relatively high sorting efficiency, which cannot be achieved using existing methods and technologies. 

### 4.4. Characteristic of Impact AE Frequency Response Coefficient and Sound Velocity in Solid Material

In this research we have discovered that the impact AE frequency response normalized coefficient *k_C_* has m/s units, the same with the unit of velocity. In Equation (11), the units of ρ, *E*, *b*, *h* and *f* are known and the Poisson’s ratio *v* is dimensionless. $\alpha_{m,n}$ is a dimensionless coefficient. Considering the sound velocity, i.e., the pressure wave (P-wave) velocity *V_p_* in an infinitive solid can be written as:

(20) $$V_{p}=\sqrt{\frac{E(1-v)}{\rho (1+v)(1-2v)}}$$

Combining equations (11) and (20) the impact frequency response could be further written as:

(21) $$f_{m,n}=\alpha_{m,n}V_{p}\frac{\sqrt{1-2v}}{1-v}\frac{h}{b^{2}}$$

and:

(22) $$k_{C}=\alpha_{m,n}V_{p}\frac{\sqrt{1-2v}}{1-v}$$

For given materials, the *V_p_* and the Poisson’s ratio *v* are constants. Hence the coefficient *k_C_* of impact AE frequency response is a function of the pressure wave velocity and the Poisson’s ratio of specific vehicle plastic materials. Further the impact AE frequency response *f* should also be a function of *V_p_* and *v*. Further research work could also focus on this topic.

## 5. Conclusions

Through this research a novel acoustic-visual sensor-based sorting method for black and dark dyed ELV plastic material recognition and recycling by using impact acoustic emission and 3D visual sensors has been established. 

Dynamic models of the scrap impact AE resonant frequency response for two commonly used vehicle plastics, PP and ABS, as well as their modified materials PP/EPDM and ABS/PC have been established and tested. PP- and ABS-based vehicle plastic materials were separated completely except for several abnormal impacts, but the separation efficiency between pure and modified plastics still has limitations. For mixtures of ABS and ABS/PC, theoretically the separation efficiency could achieve a 75% maximum value and for mixtures of PP/EPDM the separation efficiency could achieve a 50% maximum value, respectively, in the particle size range 14–23 mm. The actual separation rates were 39.2% for PP, and 41.4% for PP/EPDM scraps and 62.4% for ABS, and 70.8% for ABS/PC scraps with diameters ranging from 14 mm to 23 mm. In the diameter range of 8–13 mm, only 25% of PP, and 27% of PP/EPDM scraps as well as 43% of ABS, 47% of ABS/PC scraps were finally separated, which was less than the theoretical efficiency. The results indicated that this method can achieve a higher effective sorting efficiency for different kinds of black vehicle plastics than existing methods, which could significantly reduce the amount of ASR for disposal and produce secondary plastic materials, but there are still difficulties in distinguishing between pure and modified plastics. The impact AE frequency response features of pure and modified plastics are located near to each other. In order to achieve high recognition and separation accuracy, we need to appropriately reduce the recognition efficiency. Hence how to find appropriate crushed particle size ranges of ELV plastics is an important theme for future study. Another limitation was that the small particle size generated inadequate signal strengths. This limitation could be optimized by increasing the falling height for impact and meanwhile optimizing the crushing parameters to decrease the small size fractions. Of course, more sensitive hardware could also be implemented in future research work.
